# Determination of the optimal connector length to enhance stability of backbone‐circularized granulocyte colony‐stimulating factor

**DOI:** 10.1002/2211-5463.13692

**Published:** 2023-08-24

**Authors:** Yosuke Yasuzawa, Risa Shibuya, Yukako Senga, Takamitsu Miyafusa, Shinya Honda

**Affiliations:** ^1^ Department of Computational Biology and Medical Sciences, Graduate School of Frontier Sciences The University of Tokyo Japan; ^2^ Biomedical Research Institute National Institute of Advanced Industrial Science and Technology (AIST) Tsukuba Japan; ^3^ Bioproduction Research Institute National Institute of Advanced Industrial Science and Technology (AIST) Tsukuba Japan

**Keywords:** backbone circularization, G‐CSF, protein engineering, protein stabilization, stability prediction

## Abstract

Improving protein stability is important for industrial applications, and one promising method for achieving this is backbone circularization. As connector length affects stability, predicting and elucidating a more stable connector length is necessary for development of the backbone circularization method. However, the relationship between connector length and protein stability has not been completely elucidated. Here, we determined the most stable connector length for granulocyte colony‐stimulating factor by changing one residue at a time to produce connector length variants and then measuring their thermal stability. Analysis of the local structures obtained from the predicted structures of the circularized variants revealed that an approach using helix length, dihedral backbone angle, and number of unbonded hydrogen bond donors and acceptors is suitable for identifying connector lengths with higher stability.

## Graphical abstract

Backbone circularization enhances protein stability for industrial use. We determined the optimal connector length for granulocyte colony‐stimulating factor by evaluating the thermal stability of variants. Analysis of alphafold2‐predicted structures showed that incorporating factors such as helix length, backbone dihedral angle, and free hydrogen bond donors and acceptors practically facilitates efficient and expedited stability design.

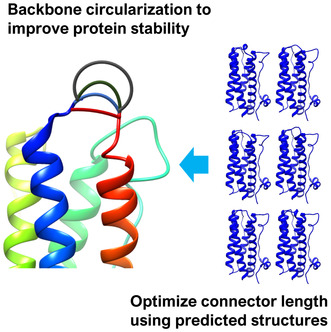

**Abbreviations**
CD: circular dichroism
DSC: differential scanning calorimetry
DSF: differential scanning fluorometry
G‐CSF: granulocyte colony‐stimulating factor
*K*_D_
: equilibrium dissociation constant
*k*_off_
: dissociation rate constant
*k*_on_
: association rate constant
PBS: phosphate‐buffered saline
PDB: Protein Data Bank
SPR: surface plasmon resonance
*T*_m_
: denaturation midpoint temperature
Δ*G*
: Gibbs free energy change

Biopharmaceuticals are superior to small‐molecule drugs owing to their higher target specificity and affinity [1]. However, proteins can be easily denatured. The resulting denatured proteins may reduce drug efficacy and cause undesirable immune responses. Consequently, biopharmaceuticals are stored and transported carefully under strict temperature control [2]. Therefore, there is a strong need to improve the long‐term storage stability of biopharmaceuticals under thermal, mechanical, and light stresses [3]. Hence, the development of versatile technologies is needed to enhance the structural stability of proteins by means of improving their storage stability or thermal stability.

There are several ways to increase the structural stability of proteins [4, 5, 6, 7], and there is no universal method. The introduction of disulfide bonds and backbone circularization limit the degree of conformational freedom of a protein in the unfolded state, thereby stabilizing the protein [8]. While disulfide bonds can be introduced between any adjacent chains, an increase in the number of cysteine residues can lead to disulfide scrambling or steric hindrance due to large sulfur atoms, thereby making it difficult to specify suitable sites for their introduction. In addition, the possible solvent conditions are limited in reductive conditions. In contrast, backbone circularization, in which the N and C termini of a protein are connected through an amide bond, can be employed under various solvent conditions. Several methods are available for backbone circularization [9, 10]. The split intein method requires shorter flanking sequences for circularization than the other methods, such as those using sortase. It is easier to purify the products while using this method (i.e., the circularization reaction is spontaneously completed during the expression and/or purification) [11].

The structural stability of a protein is defined as the difference in Gibbs free energy Δ*G* (= Δ*H* − *T*Δ*S*) between the folded and unfolded states [12]. As circularization limits the possible conformations of a denatured protein and decreases the entropy of the unfolded state, it leads to a larger Δ*G* and increases the structural stability. In backbone circularization, a shorter connector length decreases the entropy of the unfolded state and increases the structural stability. However, if the connector length is too short, the protein molecule may become unstable [13]. Therefore, optimization of the connector length is necessary to obtain more stable proteins. Zhou [14] assessed the relationship between connector length and stability assuming that a protein is a worm‐like chain. They have reported that maximum stability was achieved at a certain connector length based on their theory. In this method, only the distance between the two ends of a linear protein and number of peptide bonds present in the connector were considered as variables to calculate the stabilization energies. Using this simple method, the stability maxima of PIN1 WW domain, a small 34‐residue single‐domain protein, were well predicted. However, at lengths outside the maxima, the accuracy of the prediction was reduced. In addition, there are a few examples of the application of this method. Therefore, further understanding of the relationship between the connector length and stability is required to expand the use of backbone circularization for developing more stable proteins.

Granulocyte colony‐stimulating factor (G‐CSF) is a cytokine that promotes neutrophil differentiation and proliferation. Recombinant G‐CSF is used as to treat for neutropenia. Wild‐type G‐CSF is known to have low structural and colloidal stability and is prone to aggregation under neutral conditions [15, 16]. Therefore, an improvement in the storage stability of G‐CSF preparations is required [17]. G‐CSF has a 4‐helix bundle structure, and its N and C termini are close together [18]. In the crystal structure of the complex of human G‐CSF with the receptor [Protein Data Bank (PDB) ID: 2D9Q], six N‐terminal residues were not determined because of low electron density, indicating that the N‐terminal segment is flexible. Therefore, backbone circularization connecting the N terminus to the C terminus is expected to have little influence on the entire structure of G‐CSF. Furthermore, as the two receptor‐binding sites of G‐CSF are structurally distant from the N and C termini, circularization of G‐CSF is expected to have little influence on receptor affinity. In fact, circularized G‐CSF has already been synthesized by methods using sortase [9] or split intein [19], and it has been found that the stability of G‐CSF was improved while maintaining its original structure and receptor affinity. In particular, several variants of circularized G‐CSF with different connector lengths developed by trimming the N‐terminal segment of wild‐type G‐CSF showed significant improvement in thermal stability [19, 20] as well as low aggregation propensity [21]. In these studies, informatic analysis was conducted using the PDB database to determine the connector lengths of the variants.

Previous studies have shown that backbone circularization improves the thermal stability of G‐CSF while preserving its activity [19, 20]. However, only a limited number of variants with different connector lengths were examined. Further, the relationship between connector length and protein stability has not been completely investigated. A clear understanding of the relationship between the connector length and stability would be useful for promoting the use of backbone circularization method to improve protein stability. Furthermore, if the stability of circularized variants can be estimated based solely on the predicted structure obtained by informatics methods only, it would further expand the usefulness of backbone circularization in protein engineering.

In the present study, we aimed to determine the optimum connector length of backbone‐circularized G‐CSF that maximizes its stability and to clarify the relationship between the connector length and stability. We prepared a series of variants in which the connector length was changed by one residue and analyzed their structural stability. Receptor affinities and cell proliferation activities were also analyzed. Furthermore, we attempted to develop a simple stability prediction method by comparing experimental results with energy calculations based on the predicted structures.

## Materials and methods

### Construction of plasmid vectors

**Fig. 1:**
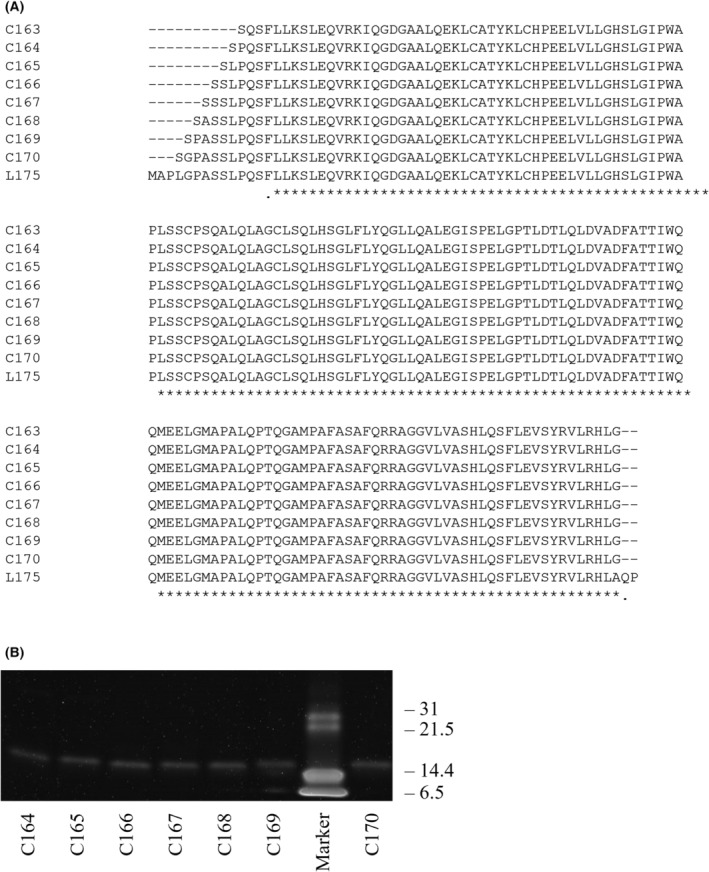
Sequence and purification of G‐CSF connector‐length variants. (A) Amino acid sequences of G‐CSF variants. (B) SDS/PAGE for isolated G‐CSF variants. The samples were analyzed after the final ion‐exchange purification. A reference SDS/PAGE standard (Broad) was used as the marker.

The name codes of the variants were given based on C for circularization, followed by the number of total amino acids (e.g., C163). The connector length was equal to the number minus 161. Plasmid vectors of linear G‐CSF (L175) and some circularized variants (C163, C166, C170, and C177) were prepared in a previous study [19]. Vectors for the C164, C165, and C167 were generated from C166 by inverse PCR‐based site‐directed mutagenesis using the KOD‐Plus‐Mutagenesis Kit (TOYOBO, Osaka, Japan). C168 and C169 variants were obtained from C170. Nucleic acid sequences of plasmid vectors were verified by Sanger sequencing [22]. The amino acid sequences of the variants are listed in Fig. 1A.

### Protein expression and purification

Protein expression and purification were performed as in the previous study [19]. After transforming *Escherichia coli* BL21 (DE3) with the plasmid vectors, colonies were picked and pre‐cultured at 37 °C overnight, and then, they were incubated at 37 °C in 1 L LB medium containing 100 μg·mL^−1^ ampicillin. When the optical density at 600 nm reached approximately 1.0, isopropyl β‐d‐1‐thiogalactopyranoside (IPTG) was added (final concentration, 0.5 mm). Cells were incubated for 3 h after induction and harvested by centrifugation. The pellets were dissolved in 25 mL PBS (137 mm NaCl, 8.1 mm Na_2_HPO_4_, 2.68 mm KCl, 1.47 mm KH_2_PO_4_, pH 7.4) containing 1% (w/v) sodium deoxycholate (DOC). The suspension was sonicated for 15 min. The pellets were collected by centrifugation. The pellets were washed (resuspended and centrifuged) in three steps using buffers containing 50 mm Tris–HCl (pH 8.0), 5 mm ethylenediaminetetraacetic acid (EDTA), and three different wash reagents [(1) 2% (w/v) Tween20, (2) 1% (w/v) DOC, and (3) 1 m NaCl]. The washed inclusion bodies were solubilized in a buffer containing 50 mm Tris–HCl (pH 8.0), 5 mm EDTA, and 6 m guanidine hydrochloride at room temperature. The supernatants were concentrated to approximately 1 mL by ultrafiltration and then diluted into 20–25 volumes of refolding buffer containing 50 mm Tris–HCl, 2 mm EDTA, 400 mm L(+)‐arginine hydrochloride, 1 mm reduced glutathione, and 0.1 mm oxidized glutathione. After equilibration in a buffer containing 20 mm Tris–HCl (pH 8.0), purification was performed.

All circularized variants were purified in three steps. First, we employed anion‐exchange chromatography using a HiTrap Q HP column (GE Healthcare, Chicago, IL, USA). Second, the fractions containing G‐CSF were concentrated up to approximately 3 mg·mL^−1^ and subjected to gel‐filtration chromatography using a Superdex 75 10/300 GL column (GE Healthcare) equilibrated with 20 mm Tris–HCl (pH 8.0) and 500 mm NaCl. Subsequently, the fractions of G‐CSF were dialyzed in a buffer containing 20 mm Tris–HCl (pH 8.0) and purified using a MonoQ 5/50 column (GE Healthcare). Fractions containing G‐CSF were concentrated by ultrafiltration. The purification status was monitored using chromatogram. Protein concentration was determined using ultraviolet absorbance at 280 nm [23]. SDS/PAGE was used to confirm that the desired products were obtained (Fig. 1B), and the ratio of the circular product to the linear byproduct was calculated (Fig. S1).

### Circular dichroism (CD)

The secondary structure of the proteins was investigated by measuring the circular dichroism (CD) spectrum in the far‐UV region using a J‐805 spectropolarimeter (JASCO, Tokyo, Japan). The proteins were equilibrated with PBS and adjusted to a concentration of 100 μg·mL^−1^. The ellipticity from 260 to 195 nm was measured at 20 °C (scanning speed: 1 nm·s^−1^, path length: 0.1 cm). Ellipticity was first measured in units of degrees, which was converted to molecular ellipticity per mole of the residue used for analysis.

The disruption of the secondary structure with heating was investigated by continuously measuring the ellipticity. The CD melting curves were recorded using the same equipment described above. The ellipticity at 222 nm was monitored during heating up from 10 °C to 90 °C at a rate of 1 °C·min^−1^. The thermodynamic parameters of the proteins for equilibrium unfolding were obtained through fitting calculation using Formula 1 based on a two‐state transition model [24].

### Differential scanning fluorometry (DSF)

The DSF assay was performed using Tycho NT.6 (Nanotemper Technologies, Munich, Germany). After equilibration with PBS, the concentration of the samples was adjusted to 100 μg·mL^−1^. Then, the solutions were aspirated using a capillary and placed in the instrument. Temperature was increased from 35 °C to 90 °C in 3 min. Excitation light at 280 nm was applied, and the intrinsic fluorescence intensities of tryptophan at 330 and 350 nm were recorded. The ratio of fluorescence intensities at 330 to 350 nm corresponded to the solvent exposure of tryptophan. The ratio of fluorescence intensity at the two wavelengths plotted against temperature and melting midpoint temperature was obtained through fitting calculations using the same model as that used for CD (Formula 1).

### Differential scanning calorimetry (DSC)

The thermal stabilities of C166 and C168 were confirmed using Nano DSC (TA Instruments, New Castle, DE, USA). Samples were dialyzed into a buffer containing 10 mm acetate (pH 4.0) and 0.004 mg·mL^−1^ Tween20; their concentrations were subsequently adjusted to 500 μg·mL^−1^. The dialysis solution was used as the reference buffer. Temperature was raised from 20 °C to 90 °C at a rate of 1 °C·min^−1^ and then cooled down from 90 °C to 20 °C at the same rate; this cycle was repeated twice. The obtained data were fitted using a two‐state model, and *T*_m_ and Δ*H* were calculated.

### Surface plasmon resonance (SPR)

The binding of G‐CSF variants to the G‐CSF receptor was measured by surface plasmon resonance (SPR) using a Biacore T200 (GE Healthcare). First, Protein A was immobilized on the CM5 sensor chip (GE Healthcare) via amine coupling in an immobilizing buffer containing 10 mm sodium acetate (pH 5.0). Purchased Fc‐chimera G‐CSF receptor was injected and captured via Protein A in running buffer (10 mm HEPES, 0.05% Tween20, 150 mm NaCl, pH 7.4). To measure the interaction with the receptor, each G‐CSF variant was sequentially injected into a flow cell in a single cycle manner at increasing concentrations and a flow rate of 30 μL·min^−1^. To measure the next variant, the sensor chip was regenerated by removing the G‐CSF receptor using a solution containing 10 mm glycine‐HCl (pH 2.0). Kinetic constants, including *k*_on_, *k*_off_, and *K*_D_, were calculated using software supplied with the instrument (Biacore T200 Control Software and Biacore T200 Evaluation Software, version 1.0, GE Healthcare).

### Cell proliferation assay

NFS‐60 cells, which proliferate in accordance with the G‐CSF concentration, were used to measure the cell proliferation activity of G‐CSF variants [25]. NFS‐60 cells (ATCC CRL‐1838) purchased from ATCC (Manassas, VA, USA) were maintained in RPMI 1630 medium containing 10% fetal bovine serum (FBS) and 100 pg·mL^−1^ human G‐CSF at 37 °C and 5% CO_2_. The cells were used for proliferative activity assay after three passages. G‐CSF variants were diluted in RPMI 1630 medium containing 10% FBS and no G‐CSF, and 11 dilution series were prepared in 5‐fold steps from 30 nm to 307 am (0.3 fm). Subsequently, 50 μL of cell suspension in the medium without G‐CSF and 50 μL of G‐CSF diluent were added to a 96‐well plate, which was then incubated for 48 h at 37 °C in 5% CO_2_. Next, 20 μL of CellTiter 96 AQueous One Solution Cell Proliferation Assay (Promega, Madison, WI, USA) was added, and cells were incubated for 2 h at 37 °C in 5% CO_2_. The number of viable cells was determined by measuring the absorbance at 490 nm using an Enspire2300 (PerkinElmer, Waltham, MA, USA). EC_50_ and *E*_max_ were determined through a fitting calculation using Formula 2. Three independent measurements were performed, and significant differences were verified by *t*‐test.

### Prediction of the three‐dimensional structure and structural characterization

The three‐dimensional structure of circularized G‐CSF was predicted using alphafold2 running on Colab [26, 27]. As alphafold2 does not support backbone‐circularized proteins, virtual circular permutation variants were used to obtain the predicted structure. A virtual truncation between ^38^Thr and ^39^Tyr at the end of A‐helix was performed with reference to a previous study [28] that conducted circular permutation experiments on wild‐type G‐CSF. The input sequence is shown in Fig. S2. All parameters except for the sequence were set to default (v1.2, msa_mode: MMseqs2 Uniref+Environmental, num_models: 5, num_runs: 3, use_amber: False). Each of the five predicted structures obtained for individual variants was further analyzed. First, to confirm the validity of the predicted structure, the difference between the predicted and crystal structures was confirmed by RMSD of the backbone of G‐CSF, for which the crystal structure is known. Using ucsf chimera, we also assigned the secondary structure of the variants using ksdssp method [29], analyzed the dihedral angles of the backbone using Ramachandran plot [30], and examined the number of hydrogen bonds [31]. The hydrogen bonds in the backbone in and around the connector were then counted.

### Stability estimation

The structural stability of the variants was estimated using foldx [32] with the structures predicted using alphafold2. In foldx, ‘Repair action’ was first performed for each predicted structure, and then various energy components were calculated. The obtained energy values of each variant were compared with the value of *T*_m_ determined from CD measurements, and multiple linear regression analysis was performed to identify the factors that have a strong influence on the thermal stability of circularized proteins.

### Formulae

#### Formula 1: theoretical curve for heat denaturation analyses

$$f\left(T\right)=\left(A_{u}\times T+B_{u}\right)+\frac{\left(A_{f}\times T+B_{f}\right)-\left(A_{u}\times T+B_{u}\right)}{\exp\left(\frac{-\text{Δ}H_{m}}{R}\frac{T_{m}}{\left((T_{\text{m}}/T)-1\right)}+\frac{\Delta C_{p}}{R}\frac{1}{\left((T_{m}/T)-1-\ln\left(T_{m},/,T\right)\right)}\right)+1}$$

A_u_ and A_f_ are proportional to the temperature portion of ellipticity in the unfolded and folded state, respectively. B_u_ and B_f_ are constant portions of ellipticity in the unfolded and folded state, respectively. *T* is the absolute temperature. *T*_m_ is the midpoint temperature of denaturation. Δ*C*_p_ is the molar heat capacity change at constant pressure and Δ*H*_m_ is the enthalpy change at *T*_m_. *R* is the gas constant. [Correction added on 07 Sep, 2023, after first online publication: Formula 1 contained a typographical error and has been corrected in this version]

#### Formula 2: logistic function for EC_50_ calculations

$$\mathrm{Abs}=\min+\frac{\mathrm{Max}-\min}{1+{\left({\text{EC}}_{50}/conc\right)}^{n}}$$

Abs is the absorbance at 490 nm. Max is the maximum absorbance of the dilution series and min is the minimum absorbance of the dilution series. *n* is the constant coefficient. *conc* is the concentration of G‐CSF.

## Results

### Protein preparation

Circularized G‐CSF variants were successfully isolated after three purification steps. The main product was separated from the byproducts by final anion‐exchange chromatography using a MonoQ column. The purification status was confirmed using SDS/PAGE (Fig. 1B).

Yields varied from each variant (Table S1), indicating that the expression level and/or refolding efficiency had an impact on the final yield. Based on the SDS/PAGE results before the last purification step with MonoQ, the circularization efficiency was calculated from the ratio of cyclic to linear chains. We observed that C164 and C169, which had the lowest yields, had low circularization efficiencies exhibited high circularization efficiencies of 86–90% (Table S1).

### Secondary structure

**Fig. 2:**
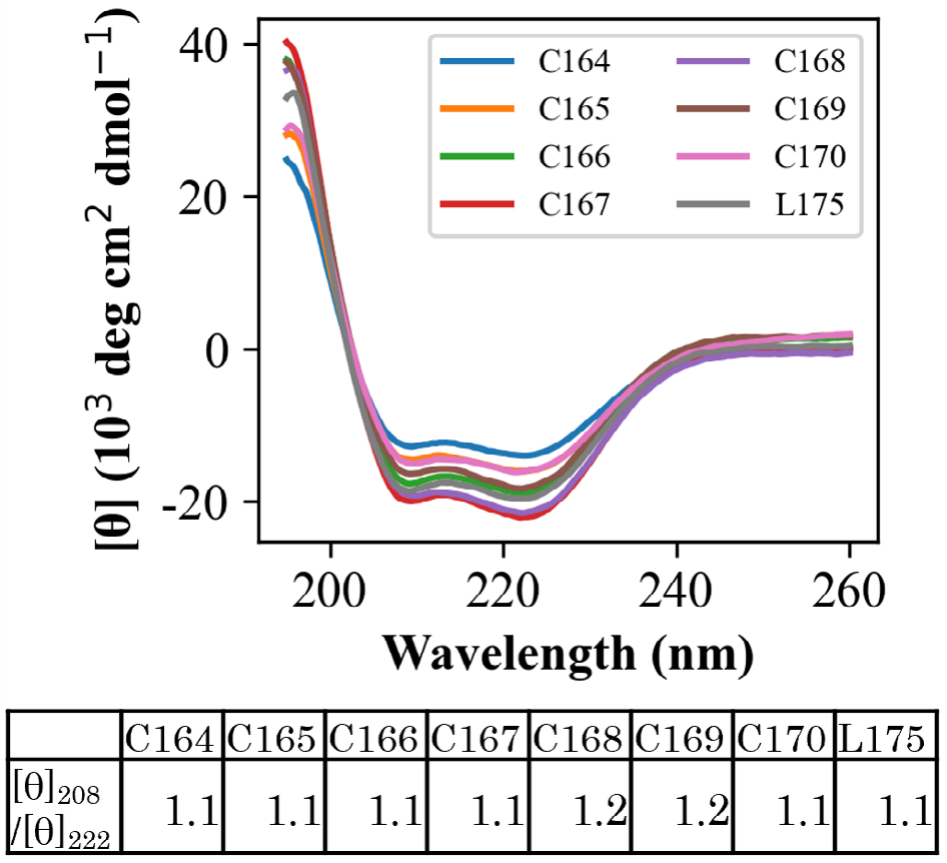
Secondary structure of G‐CSF variants as investigated by CD spectra. Data for each variant were averaged over four measurements. The lower table indicates the ratios of ellipticities at 208 and 222 nm.

Circular dichroism measurements of linear and circularized G‐CSF variants yielded similarly shaped double minimum spectra, with almost the same 222 nm/208 nm ratio (Fig. 2). This confirmed that the circularized variants retained an α‐helix‐rich structure similar to linear G‐CSF. In addition, circularized G‐CSF showed almost the same receptor affinity and cell proliferative activity as human G‐CSF, as mentioned later. These results suggested that circularized G‐CSF maintained the same three‐dimensional structure as that of linear G‐CSF.

### Thermal stability

**Fig. 3:**
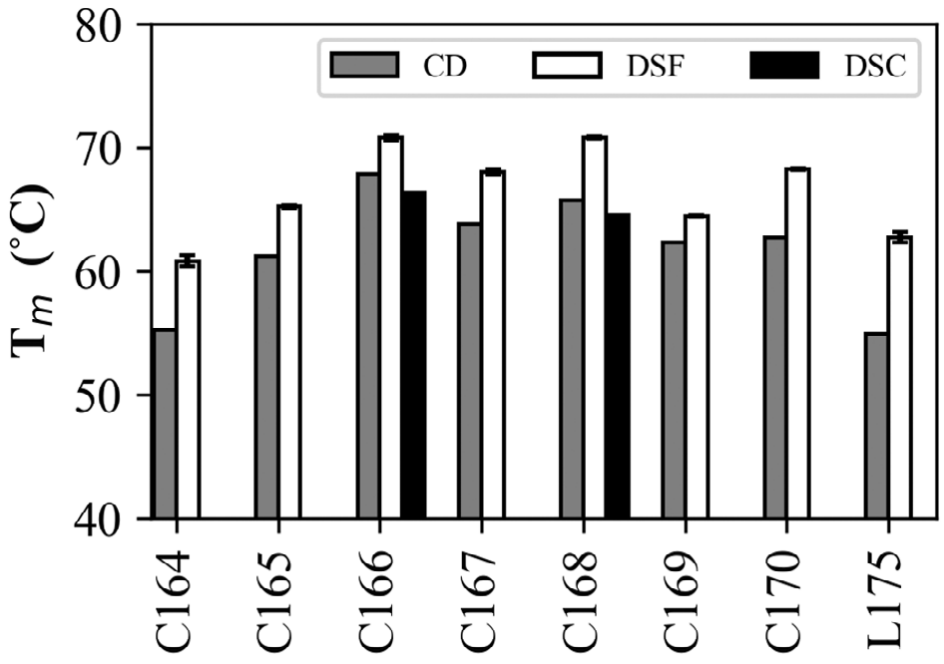
Denaturation midpoint temperature *T*_m_ of the variants as measured using CD, DSF, and DSC. DSF measurements were performed in triplicate (*n* = 3). Data are presented as the mean ± standard error. CD and DSC measurements were not repeated (*n* = 1).

Circular dichroism melting measurements were carried out and the thermodynamic parameters of the proteins for equilibrium unfolding were obtained through a fitting calculation using Formula‐1 based on a two‐state transition model. The results showed that all circularized variants, with the exception of C164, had an increased *T*_m_ compared to linear G‐CSF (Fig. 3). For variants with elevated *T*_m_, the degree of elevation was, in descending order from greatest to least, C166, C168, C167, C170, C169, and C165. In particular, C166 and C168 showed considerably elevated *T*_m_; for C166 and C168, the *T*_m_ values were 67.8 and 65.6 °C, and the increments in *T*_m_ compared to linear G‐CSF (Δ*T*_m_) were 12.9 and 10.7 °C, respectively.

Next, the peak shift of the intrinsic fluorescence of tryptophan was measured using DSF. The results showed that C166 and C168 had the highest *T*_m_ at 70.8 °C, followed by C170, C167, C165, C169, and C164 in order from highest to lowest *T*_m_ (Fig. 3). All variants showed higher *T*_m_ values than those measured using CD.

The thermal stabilities of C166 and C168, which showed high thermal stability among the variants, were further assessed using DSC. We observed that C166 had the highest thermal stability, with *T*_m_ values of 66.3 and 64.5 °C for C166 and C168, respectively (Fig. 3).

The difference in *T*_m_ between the measurement methods can be attributed to the difference in heating rate. In CD and DSC, samples were heated at 1 °C·min^−1^, while in DSF, samples were heated rapidly to 55 °C in 3 min. This resulted in a higher *T*_m_ upon analysis using DSF.

These results indicated that the variant with a connector length of five residues was the most stable in terms of disruption of the secondary structure (helix) and overall change in the tertiary structure. The variant with seven residues was the next most stable variant. Regarding the tertiary structure around tryptophan residues, the stabilities of circularized variants with five and seven residues were comparable. In terms of the relationship between the connector length and thermal stability, it was found that the stability oscillated in an even‐odd manner from the variant with a connector length of four residues to nine residues, with one residue at a time.

Notably, C164 had significantly lower thermal stability as well as lower yield and circularization efficiency than the other variants. C165 and C169, which had the next lowest thermal stabilities, had low intermediate yields after the first purification. Furthermore, C166 and C168, which showed high thermal stabilities, also exhibited higher circularization efficiencies. A positive correlation was observed between circularization efficiency and thermal stability.

### Receptor affinity

**Fig. 4:**
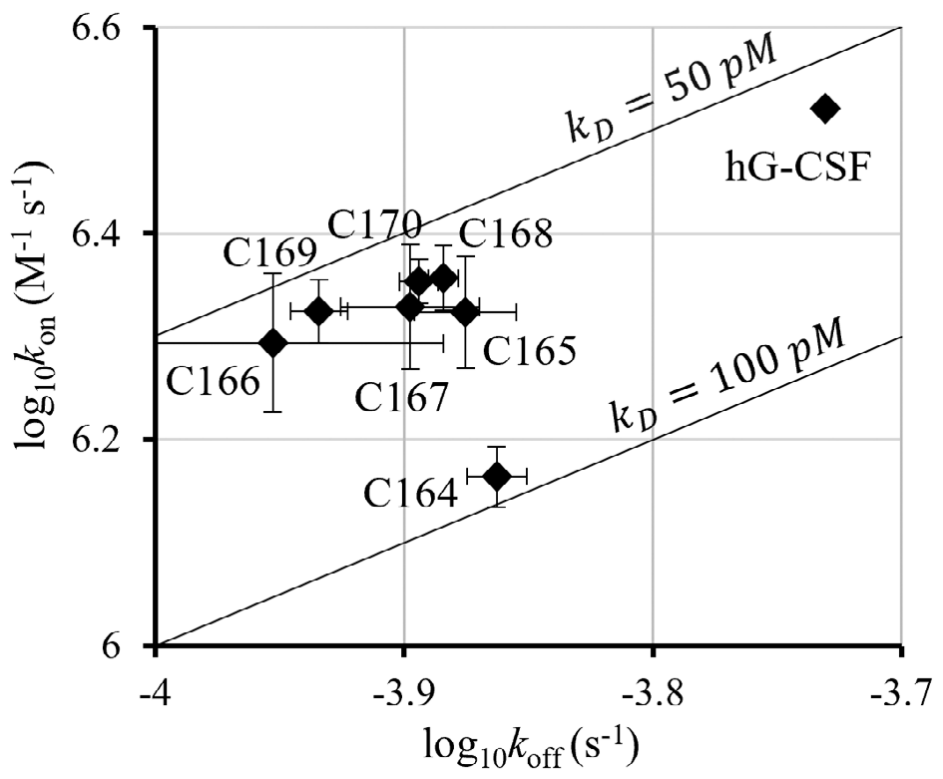
Receptor affinities of G‐CSF variants as measured by SPR. G‐CSF variants were injected into a flow cell to assess their interactions with the receptor. Kinetic constants, such as *k*_on_, *k*_off_, and *K*_D_, were calculated using the instrument software (Biacore T200 Control Software and Evaluation Software, version 1.0). Data have been presented as the mean ± standard error (*n* = 3), except for human G‐CSF.

In SPR measurements, the G‐CSF variants maintained the same receptor affinity as the human G‐CSF, except for C164 (Fig. 4). However, the dynamics of association and dissociation differed between human G‐CSF and circularized variants. Plotting each variant with *k*_on_ on the horizontal axis and *k*_off_ on the vertical axis showed that C165–C170 were clustered in one area, with C164 and human G‐CSF being the outliers (Fig. 4). Regarding human G‐CSF as the reference, C164 showed a more significant decrease in *k*_on_ than in *k*_off_, resulting in an increased *K*_D_ compared to human G‐CSF. C165–C170 showed decreases in both *k*_on_ and *k*_off_; however, the degree of these decreases was comparable, resulting in a *K*_D_ similar to that of human G‐CSF. In other words, the circularized G‐CSF showed similar affinity to human G‐CSF, but exhibited slower dynamics in terms of both association and dissociation. The receptor affinities for C163, C166, C170, C177, and linear L175 were analyzed using the same method as in a previous study [19]. Considering the present results together with those of the previous study [19], the variants with a connector length that was too short had lower thermal stability and receptor affinity. In contrast, no difference in receptor affinity was observed for the other variants with improved thermal stability, indicating that receptor affinity was not as sensitive to differences in connector length as thermal stability, except for variants with connector lengths that were too short.

### Cell proliferative activity

**Fig. 5:**
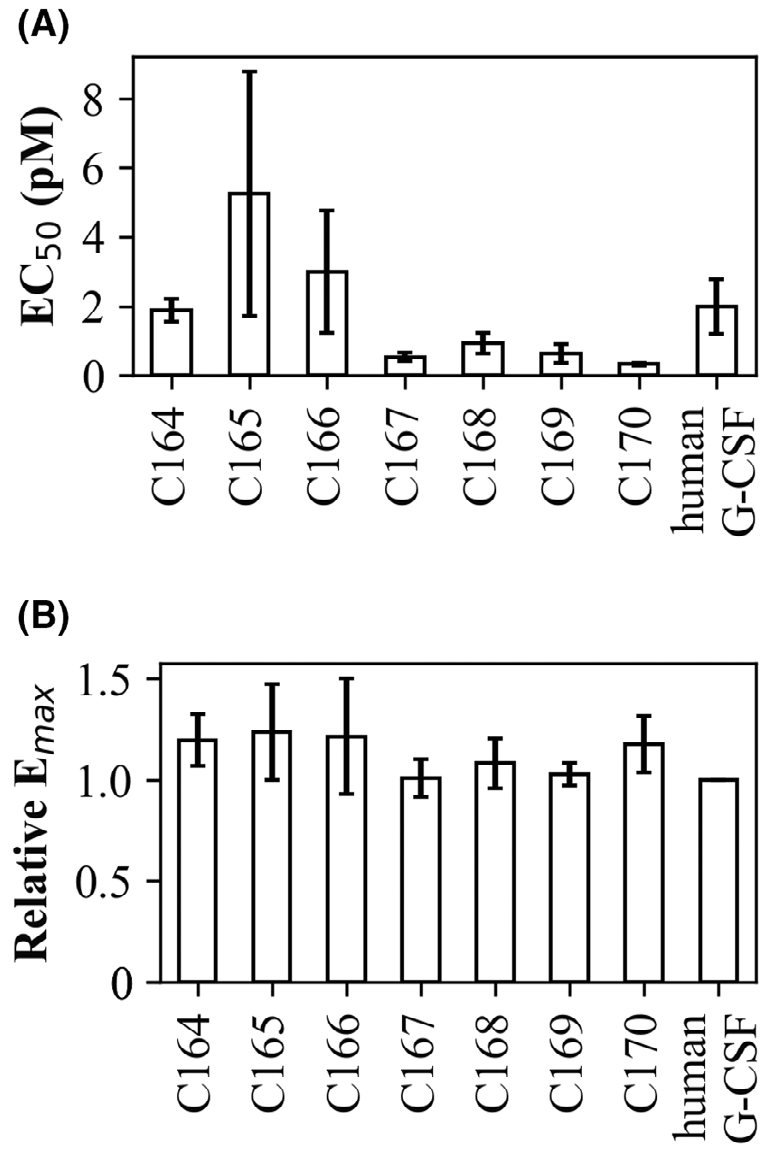
Cell proliferation activity. (A) EC_50_ of variants calculated based on Formula 2. None of the variants was significantly different from human G‐CSF. (B) Relative *E*_max_ compared to that of human G‐CSF. *E*_max_ was defined as Max in Formula‐2. In both graphs, data are presented as the mean ± standard error (*n* = 3).

The cell proliferation assay was performed using NFS‐60 cells. All circularized variants had almost the same level of *E*_max_ and EC_50_ as human G‐CSF, confirming that they maintained their cell proliferative activity (Fig. 5). No clear correlation was found between cell proliferation activity and receptor affinity, as measured by SPR. The EC_50_ values appeared to indicate a relatively high cell proliferation activity for C167–C170 and relatively low activity for C165. However, the difference was less than 10‐fold, which was comparable to the variation among the experimental trials. Therefore, no statistically significant differences in the activities of the variants were observed in the present study.

### Characterization of predicted structures

Structural prediction was performed using alphafold2 with a circular permutation sequence. Five predicted structures were obtained for each of the variants (Fig. S3). Although the global structures were consistent among the five predicted structures, the connector and loop connecting the B‐ and C‐helices formed a different structure. The global structures were consistent among all the variants. The crystal structure of C166 is known (PDB ID: 5ZO6). Therefore, the predicted structures of C166 were compared to its crystal structure. Because the RMSD of the backbone of the crystal and predicted structures of C166 was less than 1 Å, we hypothesized that the predicted structures could be used to discuss the structure–stability correlations of the circularized variants.

**Fig. 6:**
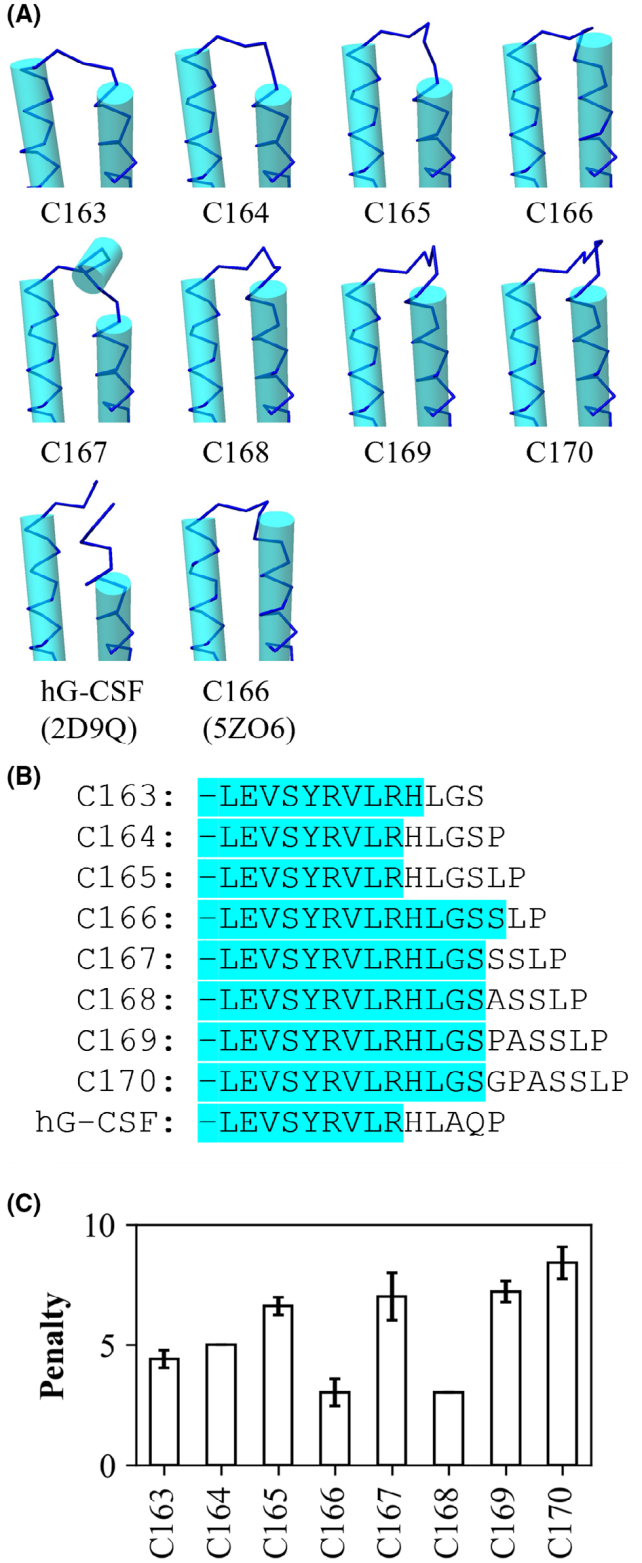
(A) Schematic of the connector structures. All structures of G‐CSF variants (C163–C170) were predicted using Alphafold2. 2D9Q and 5ZO6 are the crystal structures registered in PDB. Helix region was assigned using ksdssp. (B) Length and sequence of D‐helix. The blue characters are residues that constituted the helix in most predicted structures (3 or more out of 5). The green characters are residues that constituted the helix in some predicted structures (2 out of 5). (C) Penalty is defined as the number of unbonded donors and acceptors at and around the connector. Data have been presented as the mean ± standard error. Five structures were predicted for each variant.

Secondary structure assignment by ksdssp [19] showed that in all predicted structures, ^11^Gln to ^36^Cys formed the A‐helix. The coordinates of these helix structures also overlapped well. However, the structure of the connector and length of the D‐helix differed among the variants (Fig. 6A,B). In the wild‐type human G‐CSF (PDB: 2D9Q, crystallized in complex with the receptor), the D‐helix ends at ^169^Arg. In C163, C164, and C165 variants, the D‐helix ended at ^170^His (or ^169^Arg depending on the predicted structure). In contrast, in C166, C167, C168, C169, and C170 variants, the D‐helix was further elongated by three amino acid residues: ^171^Leu, Gly added for the intein reaction, and ^7^Ser on the original N‐terminal side.

Next, the dihedral angles of the backbone in the circularized region were determined using Ramachandran plot. Some variants had connectors with favorable dihedral angles, while others had connectors with unfavorable angles. All residues of the C166 connector were formed with acceptable dihedral angles; those of the C168 connector were formed with acceptable angles, except for Gly. In contrast, the C167 and C169 connectors had an unfavorable dihedral angle for Ser and Pro, respectively, in all the predicted structures (Fig. S4).

Finally, the hydrogen bonds formed in the backbone were analyzed. The following general trend was found in and around the connectors: the number of hydrogen bonds increased as the connectors became longer, and simultaneously, the number of unbonded donors and acceptors also increased. Nevertheless, considering their connector lengths, C166 and C168 had fewer unbonded donors and acceptors (Fig. 6C).

### Stability estimation using predicted structures

foldx was used to estimate the stability of each variant. The total energy and its components were calculated for all predicted structures (Fig. S5). Subsequently, multiple regression analysis was performed with calculated energy value as the explanatory variable and experimentally determined *T*_m_ as the objective variable. The results suggested that three energy components, namely Backbone H‐bond, van der Waals, and Solvation Hydrophobic, mainly contributed to the fluctuation of *T*_m_. We then attempted to derive a model formula for the prediction of *T*_m_ values using these three parameters; however, the results were not suitable for precise predictions (*R* ^2^ = 0.44).

## Discussion

Backbone circularization of G‐CSF was first performed using sortase by Popp *et al*. [9]. The circularization resulted in an increase in *T*_m_ from 59.6 °C to 63.2 °C. As a recognition sequence with six residues for the sortase reaction was added to the C terminus of human G‐CSF, the connector length became 16 residues. Subsequently, Miyafusa *et al*. [19] created circularized variants with shortened connector lengths of two, five, and nine residues using split intein, in which the connector length of circularized G‐CSF was determined based on statistical probability using coordinate data from proteins with known structures. That study revealed that the variant with a connector length of five residues exhibited the highest thermal stability with *T*_m_ of 69.4 °C. This is an increase of about 13 degrees from the *T*_m_ of the linear G‐CSF (56.5 °C).

Zhou [14] have reported the relationship between the connector length and stability of circularized proteins, wherein the shift in Δ*G* upon circularization (ΔΔ*G*) was calculated by considering a protein as a worm‐like chain. ΔΔ*G* was calculated using three parameters: the number of residues in the original protein, distance between the two ends of the protein, and number of residues in the connector. Using this method, we estimated the dependence of the connector length of the circularized G‐CSF on the degree of stabilization associated with circularization. The results predicted that ΔΔ*G* would be maximum at the connector length of three residues. Furthermore, a monotonic decrease in the stability was predicted as one moved away from the three‐residue connector length. Accordingly, in the present study, we prepared all circularized G‐CSF variants with connector lengths from three to nine residues, including the length predicted to be the most stable by worm‐like chain calculation, and examined their thermal stability.

The experimental results showed that the variants with a connector length of five residues had the highest thermal stability and that a variant with the shortest connector length (three residues) had the lowest thermal stability, although its thermal stability was still equivalent to that of the linear G‐CSF. These results indicate the following: first, the thermal stability exhibited a convex function with respect to the connector length. Second, the connector length at which the stability reaches its maximum is not consistent with the predictions based on the worm‐like chain model but is consistent with the prediction based on PDB statistics. Furthermore, although the worm‐like chain model predicted a monotonous decrease in the stability as the amino acid length moved away from the maximum, the stability, in fact, did not change monotonically but oscillated by one residue.

During protein preparation, a significant difference in yields was observed among the variants (Table S1). C164, which had the lowest thermal stability among the variants, showed the lowest yield. However, no clear correlation between thermal stability and yield was observed for other variants that showed improved thermal stability. Thus, it is likely that lower thermal stability leads to structural instability, which, in turn, reduces the efficiency of circularization and/or refolding, thereby resulting in lower yields. This effect is more pronounced for variants with low thermal stability, and if the thermal stability is above a certain level, a negative effect will not appear.

No correlation was observed between the thermal stability and receptor affinity, except for C164. As the circularization site is on the opposite side of the receptor‐binding site, it is reasonable that the receptor affinity was not influenced by circularization. Contrary to our expectations, interesting results were obtained for binding/unbinding dynamics. While the dissociation equilibrium constant was not considerably changed by circularization, the association and dissociation rate constants of circularized G‐CSF with the receptor were systematically changed; both were smaller than those of human G‐CSF. In other words, the rates of association and dissociation of G‐CSF with the receptor were both reduced compared to human G‐CSF. This is thought to be due to flexibility reduction upon circularization, which makes it more difficult for conformational changes to occur. However, except for dynamics, the receptor affinity and cell proliferative activity of the circularized variants were comparable to those of the human G‐CSF. Therefore, during the future development of G‐CSF‐based drugs, problems, such as reduced activity due to circularization or adverse effects on activity due to differences in connector length, are unlikely to occur.

Assignment using ksdssp method showed that the A‐helix of G‐CSF starts from ^11^Gln in the crystal structure of wild‐type human G‐CSF complexed with its receptor (PDB ID: 2D9Q). The preceding residue, ^10^Pro, acts as an N‐cap. Even in the circularized variant C166, for which the crystal structure is known (PDB: 5ZO6), the A‐helix begins at ^11^Gln, as is the case for the wild‐type G‐CSF. Furthermore, the B‐factor of the A‐helix is small in both cases, indicating that the A‐helix is rigid in the crystal. In contrast, the B‐factor of the D‐helix is relatively large in both cases, suggesting that the D‐helix is considerably flexible in the crystal.

The structures predicted by alphafold2 showed that the ends of the connector and D‐helix were different among variants, although the A‐helix was shared as a uniform structure and did not appear to be affected by differences in the connector length. The length of the D‐helix in C163–C165 is almost the same as that of wild‐type G‐CSF, whereas the length of the D‐helix in C166–C170 is three residues longer than that of wild‐type G‐CSF. Therefore, it is likely that the extension of the helix contributes to the stability of variants larger than C166. Furthermore, for C169 and C170, loosening of the helix at the D‐helix terminus was observed. This is likely caused by the burden of smoothly connecting the extra‐long connectors. Regarding backbone connections, the Ramachandran plot showed that C167 and C169 had unfavorable conformations at the connector, which may have contributed to the minor decrease in their stability. Hydrogen bond analysis of the backbone at and around the connector revealed that C166 and C168 have fewer unbonded donors and acceptors than the other variants, which could underlie the higher stability of C166 and C168 (Fig. 6C). The difference in the stabilities between C166 and C168 can be attributed to the shorter length of C166 by two residues.

Energy calculations using foldx extracted parameters correlated with variations in *T*_m_. However, the results of the present study were insufficient to infer the stability of the variants with sufficient precision. Even trivial differences in the five predicted structures of the same variant led to relatively large fluctuations in the foldx output values. Therefore, further improvements in the accuracy of the structure prediction are desired. Molecular dynamics simulations may help to improve the accuracy of the structure prediction. In future work, it would be interesting to explore whether advanced and comprehensive simulations can reduce the discrepancies between alphafold2‐predicted structures.

In the present study, the effect of backbone circularization on the stability of G‐CSF was further investigated to clarify the relationship between the connector length and stability. Stability was found to oscillate one residue at a time in an even‐odd manner. This result contradicts the theoretical calculations based on worm‐like chains, which predicted a monotonous decrease in the stability as the connector length moved away from the point of maximum stability. Further investigation also established that the circularized variant C166 with a 5‐residue long connector, which was shown to have the highest thermal stability in a previous study, was the most stable.

Recently, several new protein design methods have been developed, in which a large number of variants are usually explored by extensive computer calculations and thoroughly tested by experiments. Some variants with large changes in sequence and/or structure showed a remarkable improvement in stability [33, 34]. Compared to these methods, this study is unique in that it aims to develop a practical method that imposes the constraints of ‘minimum trials’ and ‘minimum modifications’ to achieve the goal of a short‐term development with improved stability without increasing immunogenicity, which is an essential mission in current biopharmaceutical development. This is an advantage over other studies. As backbone circularization does not require extreme changes in the protein sequence, it has an advantage in terms of antigenicity and can be used to stabilize biopharmaceuticals. It can also be used to improve the thermal stability of various industrially important proteins such as enzymes. We look forward to further improvements in engineering techniques for backbone circularization.

## Conflict of interest

Two of the authors (TM and SH) are inventors, and a patent has been obtained for the developed molecules.

### Peer review

The peer review history for this article is available at https://www.webofscience.com/api/gateway/wos/peer‐review/10.1002/2211‐5463.13692.

## Author contributions

SH designed the study and provided guidance. YY, RS, YS, and TM planned and performed the experiments and calculations. YY and TM analyzed the data. YY, TM, and SH wrote the manuscript. All authors approved the final version of the manuscript.

## Supporting information


**Fig. S1.** SDS/PAGE used for the calculation of circularization efficiencies.
**Fig. S2.** An example of a circular permutation sequence used in alphafold2.
**Fig. S3.** Predicted structures by alphafold2.
**Fig. S4.** Ramachandran plots of the predicted structures.
**Fig. S5.** Energy parameters of the predicted structures calculated using foldx.
**Table S1.** Yields and circularization efficiencies.

## Data Availability

The data that support the findings of this study are available from the corresponding author upon reasonable request.
