# Supplementary material for: Determination of the optimal connector length to enhance stability of backbone‐circularized granulocyte colony‐stimulating factor
Source: FEBS Open Bio. 2023 Aug 24;13(10):1910–21. doi: 10.1002/2211-5463.13692 (PMC10549227; doi:10.1002/2211-5463.13692)
Supplement: Supplementary file 1 — Fig. S1. SDS/PAGE used for the calculation of circularization efficiencies. Fig. S2. An example of a circular permutation sequence used in alphafold2. Fig. S3. Predicted structures by alphafold2. Fig. S4. Ramachandran plots of the predicted structures. Fig. S5. Energy parameters of the predicted structures calculated using foldx. Table S1. Yields and circularization efficiencies. [file FEB4-13-1910-s001.pdf]

Supplementary Information

Determination of the optimal connector length to enhance stability of backbone-circularized granulocyte colony-stimulating factor

Yosuke Yasuzawa[1], Risa Shibuya[1,2], Yukako Senga[2], Takamitsu Miyafusa[2,3], and Shinya Honda[1,2]

[1] Department of Computational Biology and Medical Sciences, Graduate School of Frontier Sciences, The University of Tokyo, Japan
[2] Biomedical Research Institute, National Institute of Advanced Industrial Science and Technology (AIST), Japan
[3]Bioproduction Research Institute, National Institute of Advanced Industrial Science and Technology (AIST), Japan

TABLE OF CONTENTS

Supporting Figure S1: SDS-PAGE used for the calculation of circularization efficiencies
Supporting Figure S2: An example of a circular permutation sequence used in Alphafold2
Supporting Figure S3: Predicted structures by Alphafold2
Supporting Figure S4: Ramachandran plots of the predicted structures
Supporting Figure S5: Energy parameters of the predicted structures calculated using FoldX
Supporting Table S1: Yields and circularization efficiencies

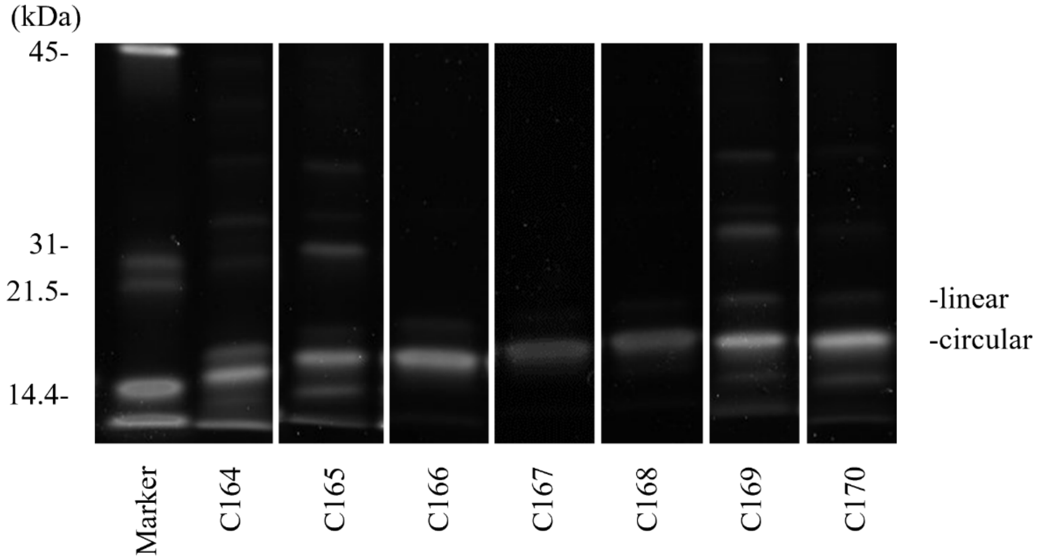

Supporting Figure S1: SDS-PAGE used for the calculation of circularization efficiencies

Circularization efficiency was calculated from the ratio of linear and circular luminance.

```
C166          SSLPQSFLLKSLEQVRKIQGDGAALQEKLCATYKLCHPEELVLLGHSLGIPWAPLSSCPS
C166_input    --------------------------------YKLCHPEELVLLGHSLGIPWAPLSSCPS
                                              ****************************

C166          QALQLAGCLSQLHSGLFLYQGLLQALEGISPELGPTLDTLQLDVADFATTIWQQMEELGM
C166_input    QALQLAGCLSQLHSGLFLYQGLLQALEGISPELGPTLDTLQLDVADFATTIWQQMEELGM
              ************************************************************

C166          APALQPTQGAMPAFASAFQRRAGGVLVASHLQSFLEVSYRVLRHLG--------------
C166_input    APALQPTQGAMPAFASAFQRRAGGVLVASHLQSFLEVSYRVLRHLGSSLPQSFLLKSLEQ
              **********************************************

C166          ------------------
C166_input    VRKIQGDGAALQEKLCAT
```

Supporting Figure S2: An example of circular permutation sequence used in Alphafold2

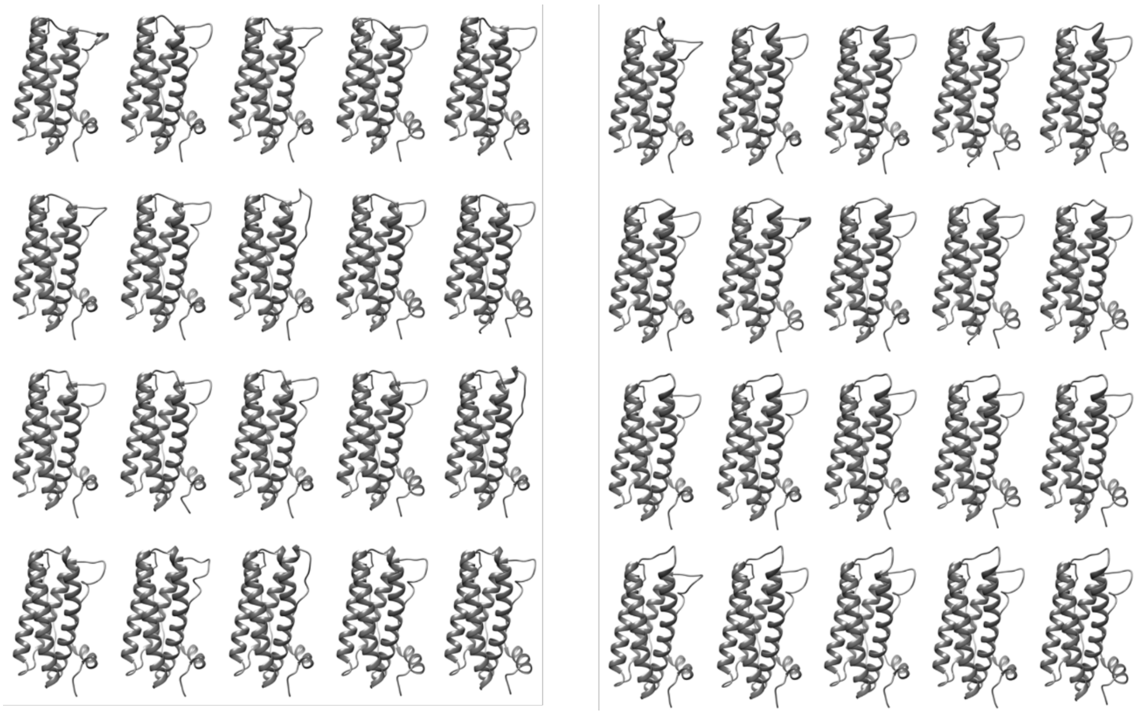

Supporting Figure S3: Predicted structures by Alphafold2

C163, C164, C165, C166 from the top left side, and C167, C168, C169, C170 from the top right side. 5 predicted structures were obtained for each variant.

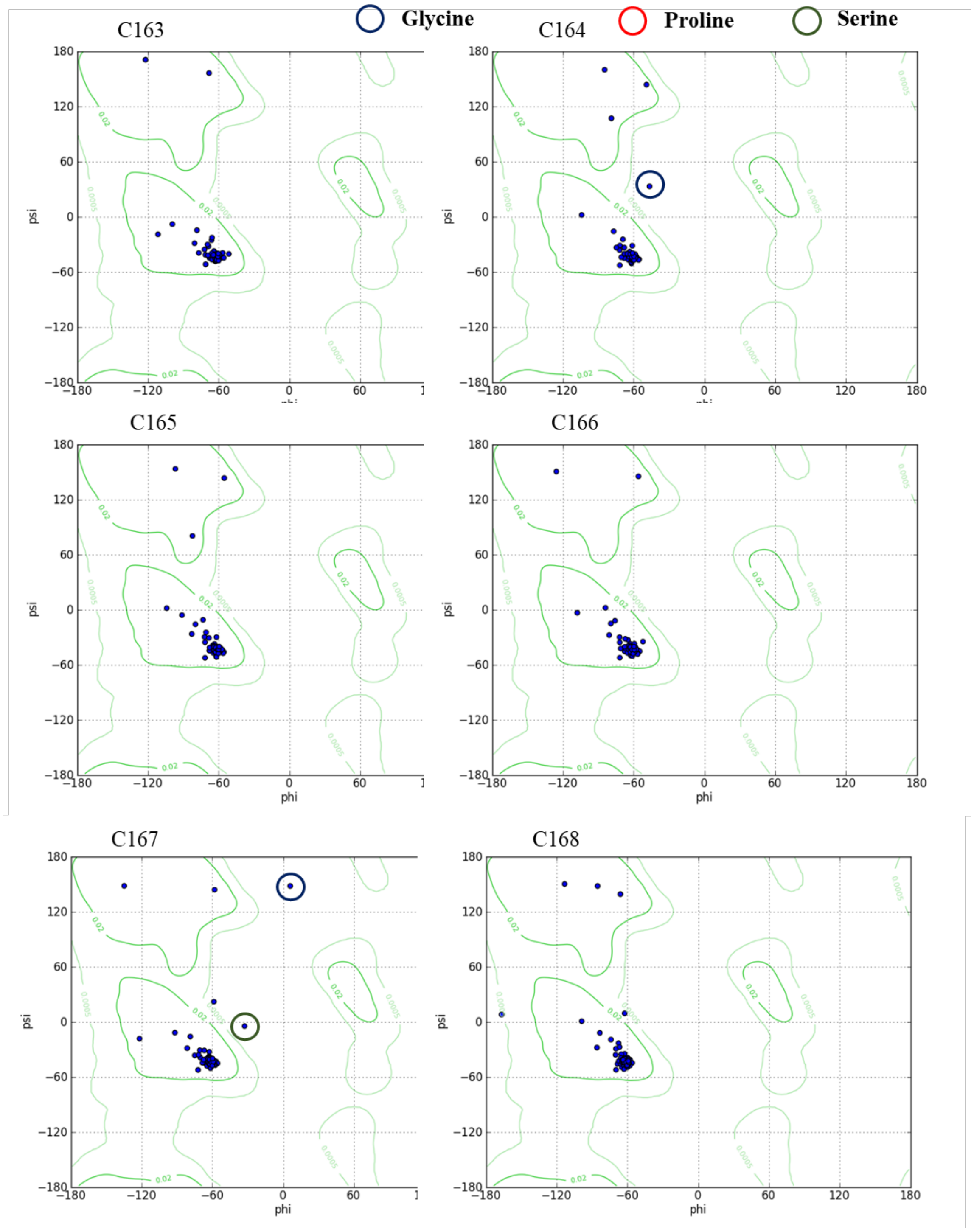
Glycine
Proline
Serine
C163
C164
C165
C166
C167
C168
psi
phi

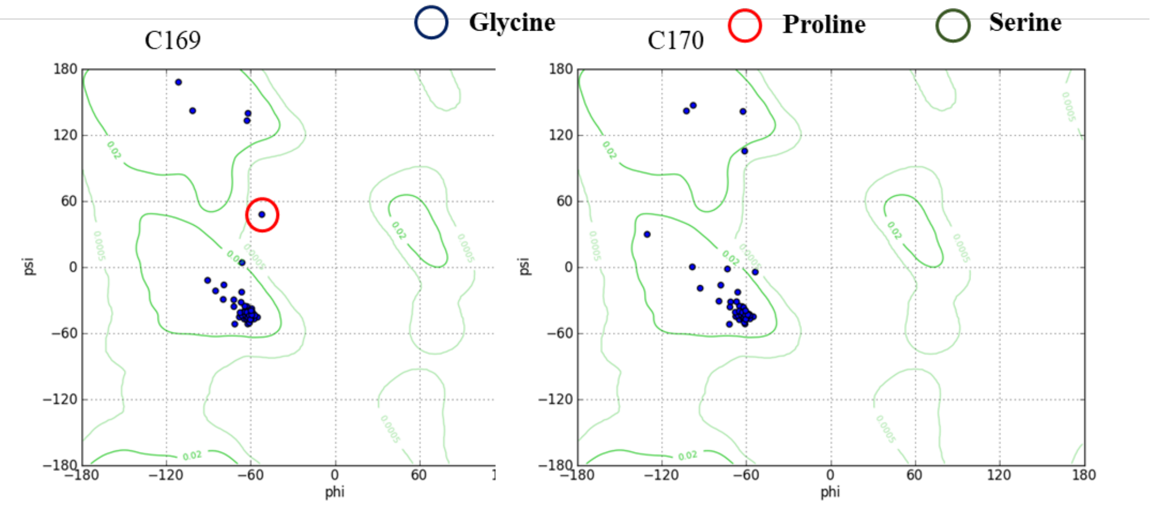

Supporting Figure S4: Ramachandran plots of the predicted structures.
Dihedral angles at and around the connector are shown.

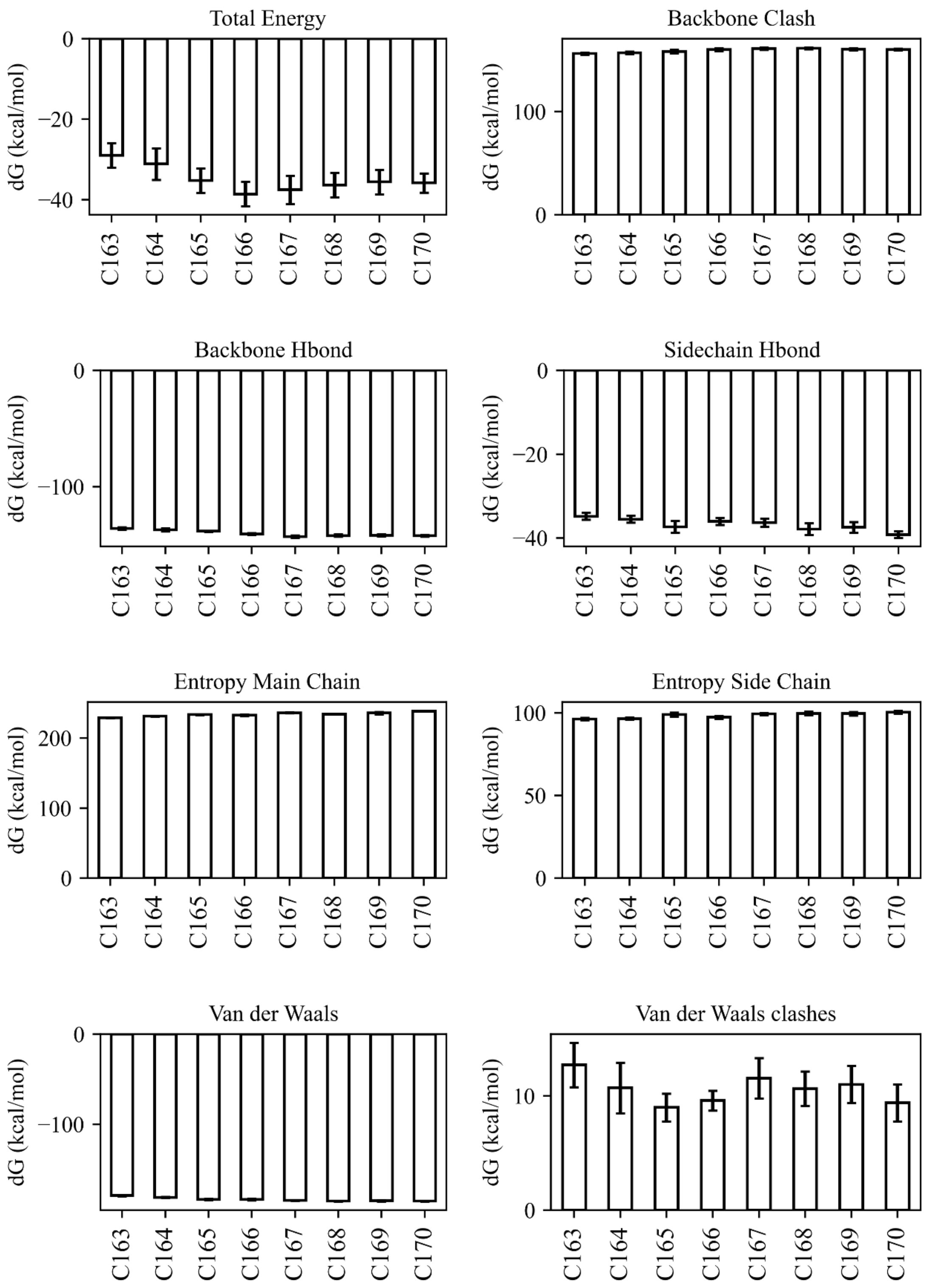
Total Energy
dG (kcal/mol)
0
−20
−40
C163
C164
C165
C166
C167
C168
C169
C170
Backbone Clash
dG (kcal/mol)
100
0
C163
C164
C165
C166
C167
C168
C169
C170
Backbone Hbond
dG (kcal/mol)
0
−100
C163
C164
C165
C166
C167
C168
C169
C170
Sidechain Hbond
dG (kcal/mol)
0
−20
−40
C163
C164
C165
C166
C167
C168
C169
C170
Entropy Main Chain
dG (kcal/mol)
200
100
0
C163
C164
C165
C166
C167
C168
C169
C170
Entropy Side Chain
dG (kcal/mol)
100
50
0
C163
C164
C165
C166
C167
C168
C169
C170
Van der Waals
dG (kcal/mol)
0
−100
C163
C164
C165
C166
C167
C168
C169
C170
Van der Waals clashes
dG (kcal/mol)
10
0
C163
C164
C165
C166
C167
C168
C169
C170

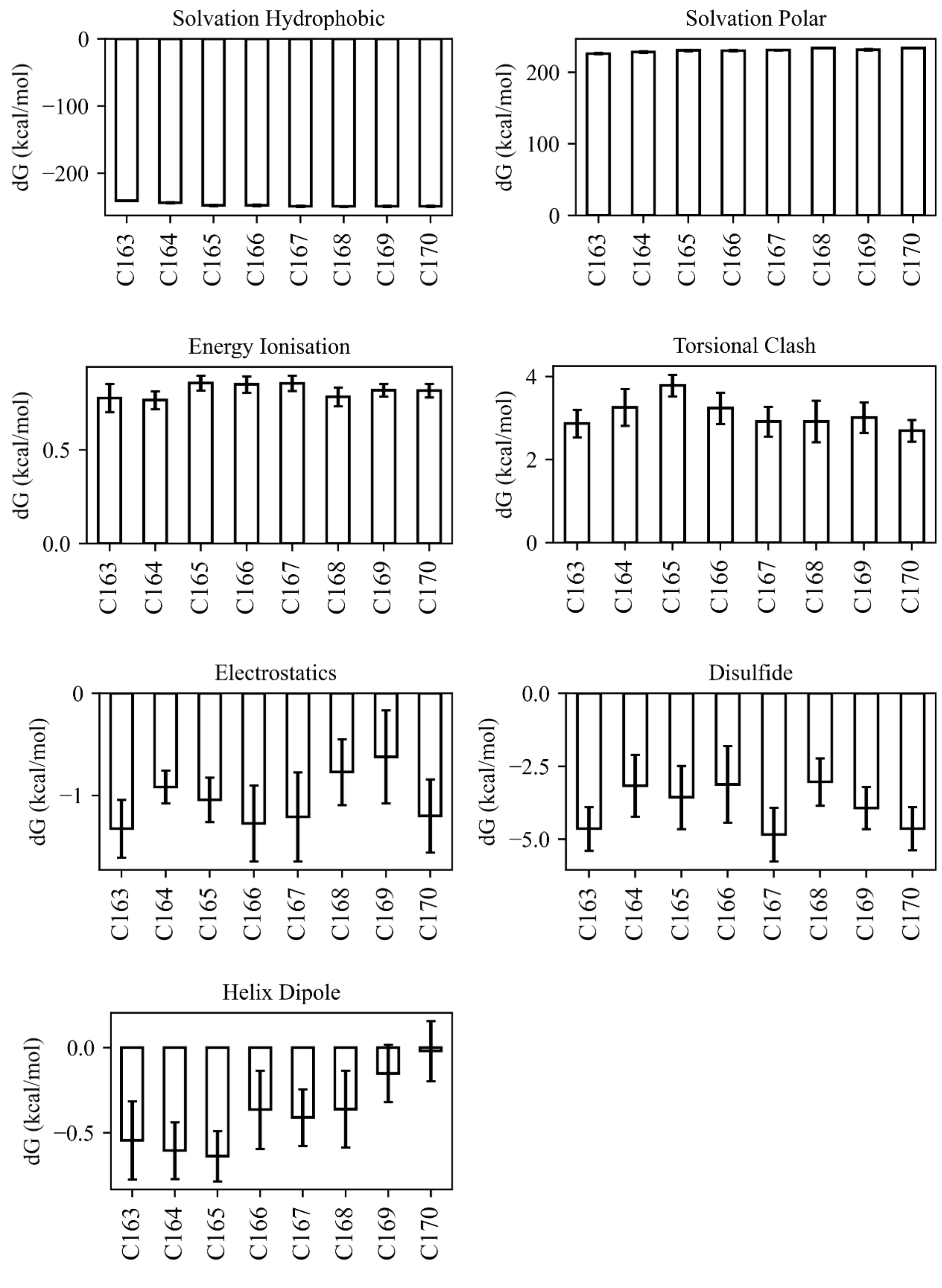

Supporting Figure S5: Energy parameters of the predicted structures calculated using FoldX.

Stability was calculated after Repair. Data are presented as the mean ±standard error (n = 5).

Supporting Table S1: Yields and circularization efficiency

	C164	C165	C166	C167	C168	C169	C170
**Yields after first purification (mg/L)**	1.21	1.24	1.55	2.08	1.55	1.04	2.08
**Final yields (µg/L)**	38.5	127	154	193	144	93.1	218
**Circularization efficiency (%)**	73.1	86.7	88.2	89.7	89.0	81.0	87.6
